# ERα-Targeting PROTAC as a Chemical Knockdown Tool to Investigate the Estrogen Receptor Function in Rat Menopausal Arthritis

**DOI:** 10.3389/fphar.2021.764154

**Published:** 2021-11-30

**Authors:** Li Duan, Xiao Xu, Limei Xu, Caining Wen, Kan Ouyang, Zigang Li, Yujie Liang

**Affiliations:** ^1^ Department of Orthopedics, Shenzhen Intelligent Orthopaedics and Biomedical Innovation Platform, Guangdong Provincial Research Center for Artificial Intelligence and Digital Orthopedic Technology, Shenzhen Second People’s Hospital, The First Affiliated Hospital of Shenzhen University, Shenzhen, China; ^2^ School of Chemical Biology and Biotechnology, Peking University Shenzhen Graduate School, Shenzhen, China; ^3^ Department of Child and Adolescent Psychiatry, Shenzhen Kangning Hospital, Shenzhen Mental Health Center, Shenzhen, China

**Keywords:** PROTACs, estrogen receptor α, MMP-13, articular cartilage metabolism, menopausal arthritis

## Abstract

Proteolytic targeting chimeras (PROTACs) is a rapid and reversible chemical knockout method. Compared with traditional gene-editing tools, it can avoid potential genetic compensation, misunderstandings caused by spontaneous mutations, or gene knockouts that lead to embryonic death. To study the role of estrogen receptor alpha (ERα) in the occurrence and progression of menopausal arthritis, we report a chemical knockout strategy in which stable peptide-based (PROTACs) against ERα to inhibit their function. This chemical knockdown strategy can effectively and quickly inhibit ERα protein *in vivo* and *in vitro*. In the rat menopausal arthritis model, this study showed that inhibiting estrogen function by degrading ERα can significantly interfere with cartilage matrix metabolism and cause menopausal arthritis by up-regulating matrix metalloproteinase (MMP-13). The results of this study indicate that ERα is a crucial estrogen receptor for maintaining cartilage metabolism. Inhibition of ERα function by PROTACs can promote the progression of osteoarthritis.

## Introduction

Menopausal arthritis is one of the most frequently encountered types of arthritic diseases (Wluka et al., 2000; Watt, 2018). This type of arthritis occurs in women who experience decreased estrogen levels, due to not only menopausal age but also artificial menopause induced by hormonal disorders or ovariectomy. Menopausal arthritis can cause cartilage degeneration and joint pain, severely restricting joint movement and thus compromising patient quality of life (Lou et al., 2016). However, the exact molecular mechanism of menopausal arthritis is still unclear.

**GRAPHICAL ABSTRACT:**
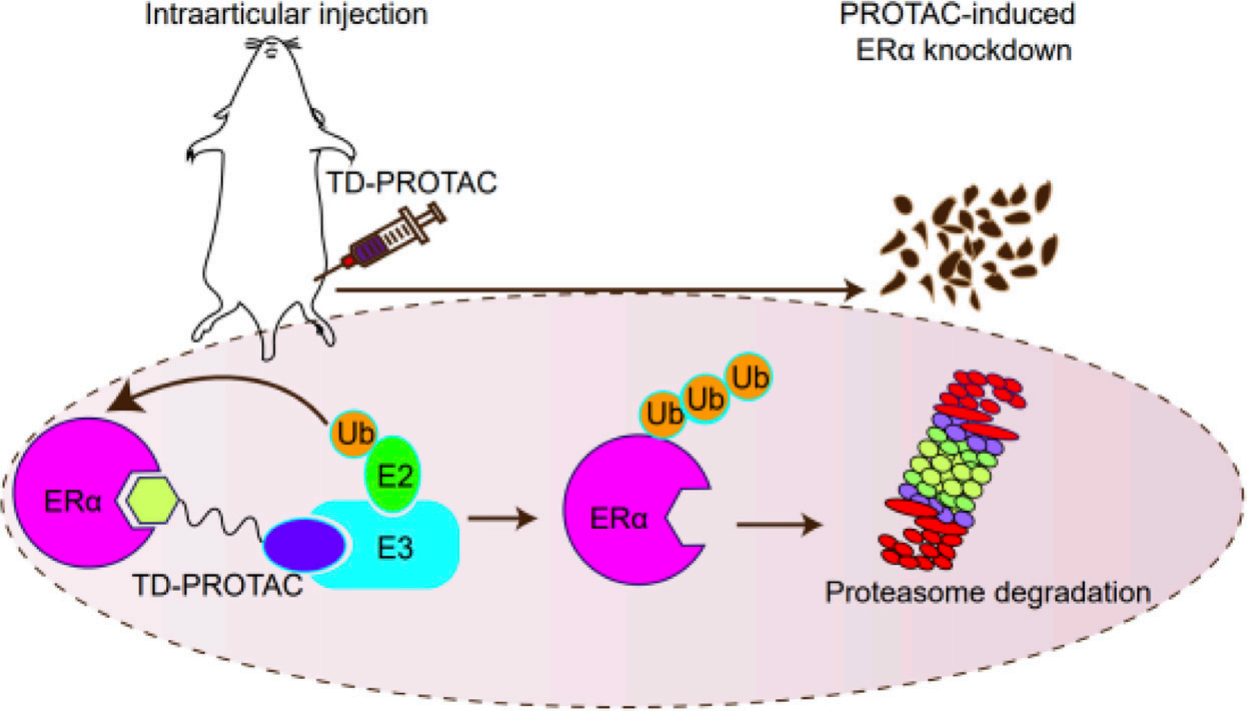
A schematic diagram of the peptide-based PROTACs to inhibit ERα function.

Previous studies have established the role of estrogen in cartilage metabolism during menopausal arthritis pathogenesis (Gokhale et al., 2004; Xu et al., 2019). However, the role of estrogen receptors (ERs) in menopausal arthritis, especially in cartilage modulation, has not been determined (Börjesson et al., 2013; Ribeiro et al., 2020). Three distinct ERs have been characterized: ERα, ERß, and ERγ. Our previous study demonstrated that estrogen acts on ERα and increases miR-140 expression levels in human articular chondrocytes. This cascade decreases matrix metalloproteinase-13 (MMP-13) expression levels (Liang et al., 2016). To date, there have been no *in vivo* studies about the effect of ERα function on menopausal arthritis progression.

Knockout the specific gene is one of the common methods to study the function. Most used knockout methods, such as transcription activator-like effector nucleases (TALEN), Cre-LoxP, clustered regularly interspaced short palindromic repeats/CRISPR-associated proteins 9 (CRISPR/Cas9), etc., can knock out at the gene level, and RNA interference can act on the transcription level (Elbashir et al., 2001; Chan, 2013; Doudna and Charpentier, 2014; Sengupta et al., 2017; Wang and Sun, 2019). These methods usually need to construct animal gene knockout models, especially non-human primate models, which are not only complicated, time-consuming, and irreversible loss of function caused by gene knockout. In addition, the deletion of some unique genes leads to the death of animals in the embryonic stage, which hinders subsequent scientific research (Doudna and Charpentier, 2014). PROTAC (Proteolysis-Targeting Chimera) represents a chemical knockdown strategy that recruits E3 ubiquitin ligase and uses its own ubiquitin-proteasome system to achieve targeted specific protein degradation (Toure and Crews, 2016). A study has used PROTACs to construct a whole-body knockdown for FKBP12 protein levels in mice, rats, pigs, and rhesus monkeys, and the function of FKBP12 protein has been studied and verified in mice and rhesus monkeys. If the administration of PROTAC is stopped, the FKBP12 protein in the animal can gradually recover, which is conducive to the control of the animal model itself and is more accurate for protein function research. This method is applied to the systemic knockdown of other targets, such as Bruton’s tyrosine kinase (BTK) protein (Sun et al., 2018). Therefore, the PROTAC technology effectively supplements the current gene knockout methods and has broad application prospects because of its rapid, reversible, and controllable realization of systemic protein knockdown *in vivo* (Sun et al., 2019b).

In a previous study, our collaborator has developed stabilized peptide-based PROTACs to inhibit ERα (Jiang et al., 2018). This kind of PROTACs can selectively recruit ERα to the Von Hippel-Lindau (VHL) E3 ligase complex and degrade ERα in a proteasome-dependent manner. In this study, we established an animal menopausal arthritis model by ovariectomy and cruciate ligament transection (ACLT) in rats to set a control. The rats have undergone the ACLT operation and then were injected with PROTACs against ERα for 4 weeks to study the effect of PROTACs on osteoarthritis progression. After euthanization, the joint tissue of rats was taken and further analyzed by immunohistochemistry. The results of this study will provide evidence for the role of ERα in menopausal arthritis progression.

## Materials and Methods

**FIGURE 1:**
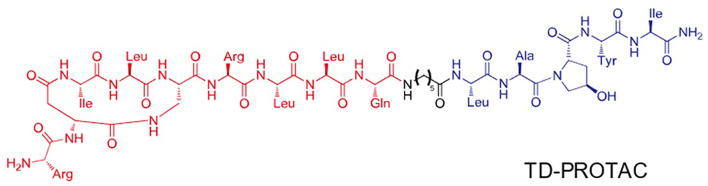
Structure-based design of TD-PROTAC. The red part indicates the sequence targeting ERα, the black part indicates the linker and the blue part indicates the ligand binding to the von Hippel-Lindau (VHL) E3 ubiquitin ligase.

The animal experiments were approved by the Ethics Committee of Shenzhen Top Biotech Co., Ltd. (No. TOP-IACUC-2020-07-0013). Six-month-old rats were purchased from Shenzhen Top Biotech Co.,Ltd. (Shenzhen, China). Twelve female rats were randomly assigned to the following groups: blank group, ovariectomized (OVX) + ACLT group, and ACLT + PROTACs group. The PROTACs were synthesized by Prof. Zigang Li’s lab from Shenzhen Graduate School, Peking University. The structure of PROTACs was shown in Figure 1.

### *In Vitro* ERα Expression Assay

To determine the role of PROTAC-mediated ERα degradation in chondrocytes, heterobifunctional peptide (TD-PROTAC)-stimulated chondrocytes were subjected to immunofluorescence imaging and western blotting.

For immunofluorescence analysis, chondrocytes were treated with TD-PROTAC for 24 h. After being washed three times with phosphate-buffered saline (PBS), the cells were fixed with 4% paraformaldehyde and then permeabilized with 0.2% of Triton X-100 for 10 min. After being blocked with 3% BSA, the cells were stained with anti-ERα antibodies. Fluorescent images of the cells were captured using an LSM800 confocal imaging system (Zeiss, Germany).

For western blot analysis, protein samples were harvested from the TD-PROTAC-treated group and control group. Then, the proteins were resolved on 12% SDS-PAGE gels and transferred to a PVDF membrane. The membranes were blocked for 1 h in 5% fat-free dry milk in PBS and then incubated with ERα-specific antibodies overnight at 4°C. Conjugated goat anti-rabbit IgG secondary antibodies (H + L) (MultiSciences, China) were added and incubated for 1 h at room temperature. Finally, the membranes were examined by an Odyssey Infrared Imaging System (LI-COR, Lincoln, NE).

### Establishment of the Menopausal Arthritis Animal Model

After the induction of anesthesia by isoflurane inhalation, the rats were placed in a prone position. A 1 cm vertical incision was made in the lower margin of the rib column. Soft tissues such as fat and muscle were separated layer by layer. Strawberry-like ovarian tissue was exposed when a white mass of adipose tissue in the abdominal cavity was separated. After tying off the ovary’s blood vessels, the ovary was dissected, and then the muscles and skin were sutured. The same procedure was performed on the opposite side. One month later, 1 ml of plasma was collected from the rats, and the estrogen level in the plasma was measured using an enzyme-linked immunosorbent assay (ELISA) kit (Biovision, Inc., Milpitas, CA, USA) according to the manufacturer’s instructions.

### Establishment of the ACLT Animal Model

For the anterior cruciate ligament exposure, the medial approach to the patella was adopted. After the skin cutting and muscle separation, the patella was turned over, and then the joint cavity was exposed. The anterior cruciate ligament was dissected using a sharp knife. The muscle skin was sutured.

### Injection and Delivery Efficiency Assay of PROTACs

One week after ACLT operation, PROTACs were injected. The kneecap was gently pressed to locate the joint capsule. Forty μM of PROTACs were slowly injected into the joint capsule perpendicular to the patella from the lateral side along the patellar edge. Then, the injection site was gently pressed for 15 s in case of leakage. PROTACs were injected once a week and lasted 4 weeks.

For *in vivo* delivery efficiency assay, FITC-peptide (2 mg of peptide in 100 μl of PBS) was intra-articularly injected. Rats were euthanized 12 h after injection of FITC-peptide, and cartilage tissue was collected. Then cartilage tissue was embedded in optimal cutting temperature (O.C.T.) compound and sectioned. The tissue sections were imaged using a confocal microscope to evaluate the delivery efficiency of FITC-labelled peptide. H33258 was used for nuclear counterstain.

### Histochemical Analysis

One week after PROTACs injection, the rats were euthanized. Knee joints were extracted and fixed in 4% neutral formaldehyde for 72 h. After being decalcified with EDTA for 45 days, the tissues were embedded in paraffin and sectioned longitudinally at a thickness of 4 μm. The slices were dewaxed and then stained with hematoxylin and eosin (HE), toluidine blue (TB), and safranin O/fast green (SO/FG).

### Immunohistochemical Analysis

The slices were dewaxed. Antigen retrieval was performed by pepsin incubation for 20 min. A 3% hydrogen peroxide solution was added to block endogenous peroxidase. After being blocked in serum, the slides were stained with primary antibodies (anti-ERα, anti-type II collagen (COL2), and anti-MMP-13) and secondary antibodies. After the antibody incubation, DAB was added. The nuclei were stained with hematoxylin and imaged under a microscope. The integrated optical density of immunohistochemical staining was quantified using the Image J software.

### Histological Score

The images of the cartilage slices were evaluated according to the modified Osteoarthritis Research Society International (OARSI) scoring system (Pritzker et al., 2006). The slices were scored by three independent researchers. Under the microscope, the slices were divided into 3 equal width zones according to the OARSI scoring table. The score of each zone ranged from 0 points to 5 points. The total score was determined by summing the scores of the three zones and ranged from 0 to 15 points.

### Statistical Analysis

Statistical analysis was performed using GraphPad Prism software, version 8.02 (GraphPad Software, USA). Statistical significance between two groups with parametric data was assessed by two-tailed t-tests. Statistical analysis comparing multiple groups with parametric data was performed by one- or two-way ANOVA with Tukey’s post hoc test. A value of *p* < 0.05 was considered statistically significant. All data are presented as the mean ± standard deviation (SD).

## Results

### Stabilized Peptide-based PROTAC-Mediated Degradation of ERα in Chondrocytes

**FIGURE 2:**
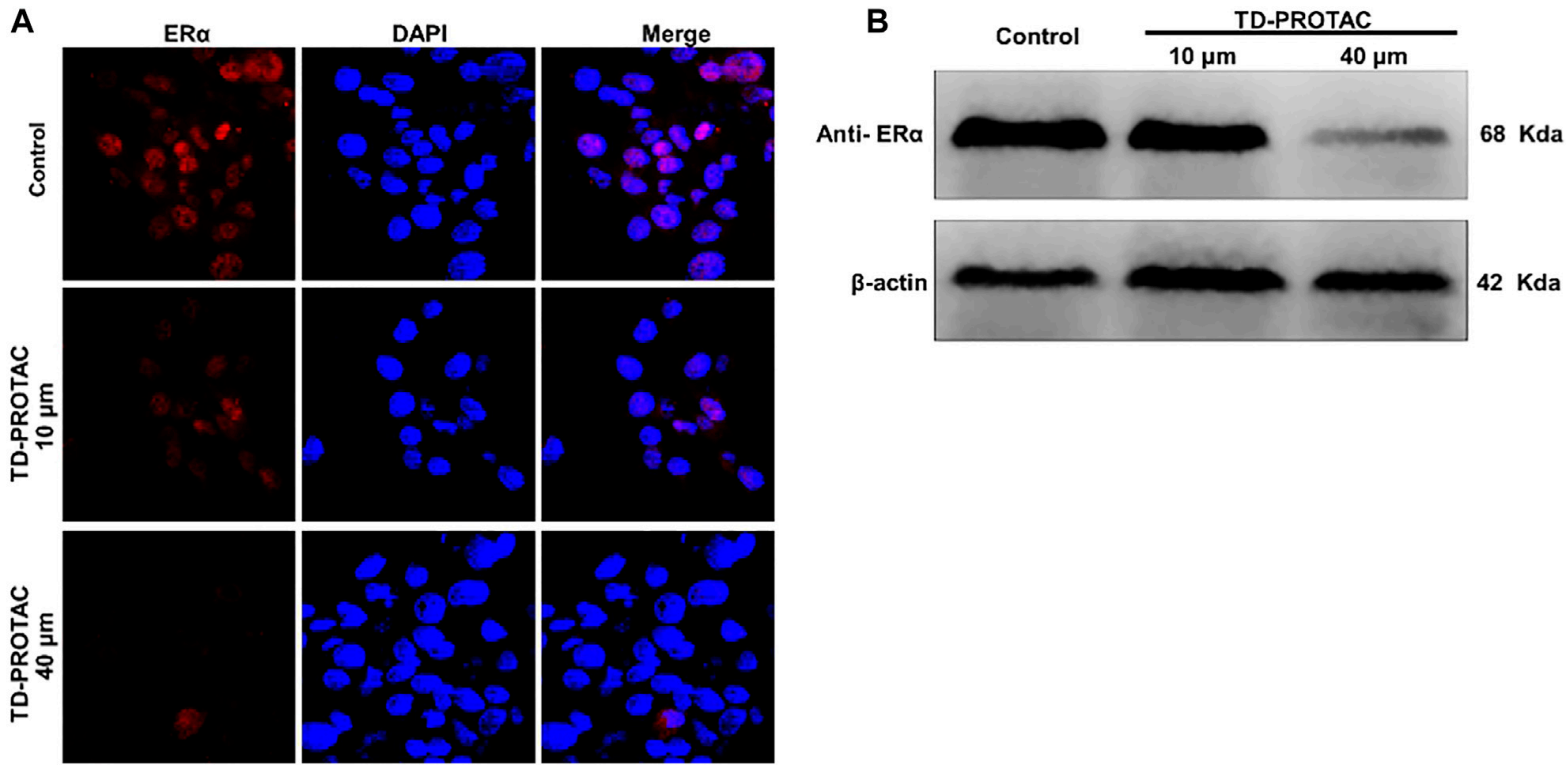
The effect of TD-PROTAC on ERα expression in chondrocytes. **(A)** Immunofluorescence analysis of ERα after staining with a secondary Cy3-conjugated antibody (red). Nuclei were stained with DAPI (blue). **(B)** Western blot analysis of ERα after treatment of cells with TD-PROTAC. Chondrocytes were treated with TD-PROTAC at the indicated concentrations for 24 h.

To evaluate the stabilized peptide-based PROTACs targeting ERα in chondrocytes, chondrocytes derived from rat articular cartilage were incubated with a heterobifunctional peptide (TD-PROTAC) for 24 h and then stained with immunofluorescent ERα antibodies. ERα colocalization with the nucleus in the untreated control group showed obviously high red fluorescence signals. Moreover, the fluorescence signals of Cy3-conjugated ERα antibodies were greatly decreased relative to those of unstimulated chondrocytes. Fluorescence signals were not detected in chondrocytes in the high concentration TD-PROTAC treatment group (40 μM) (Figure 2A).

To evaluate TD-PROTAC-mediated ERα degradation, intracellular ERα expression was also examined in chondrocytes using western blotting. Incubation with TD-PROTAC reduced the protein expression of ERα (Figure 2B).

### Estrogen Levels Were Significantly Decreased in Ovariectomized Rats

**FIGURE 3:**
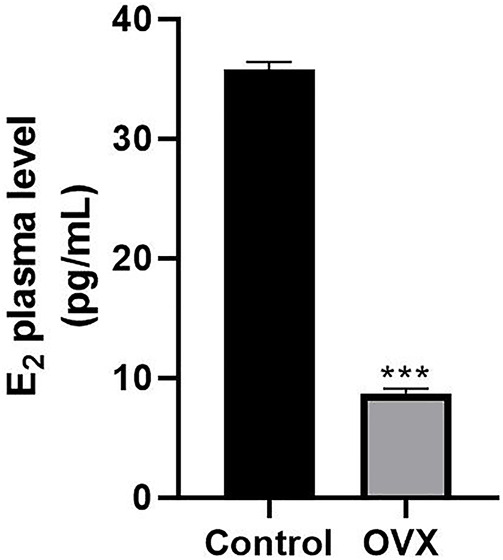
ELISA results showing changes in serum estrogen levels in rats. One month after ovariectomy (OVX), the serum levels of estrogen were compared between the OVX group and the control group (***p < 0.001).

To determine whether ovariectomy could successfully imitate estrogen deficiency conditions in rats, we compared E2 levels in healthy control rats and postmenopausal rats. The ELISA results showed that the average plasma level of E2 was 35.8 ± 0.64 pg/ml in female rats at 7 months of age. However, the E2 level was less 8.7 ± 0.39 pg/ml in postmenopausal rats (Figure 3).

### The Menopausal Arthritis Animal Model Was Successfully Established

**FIGURE 4:**
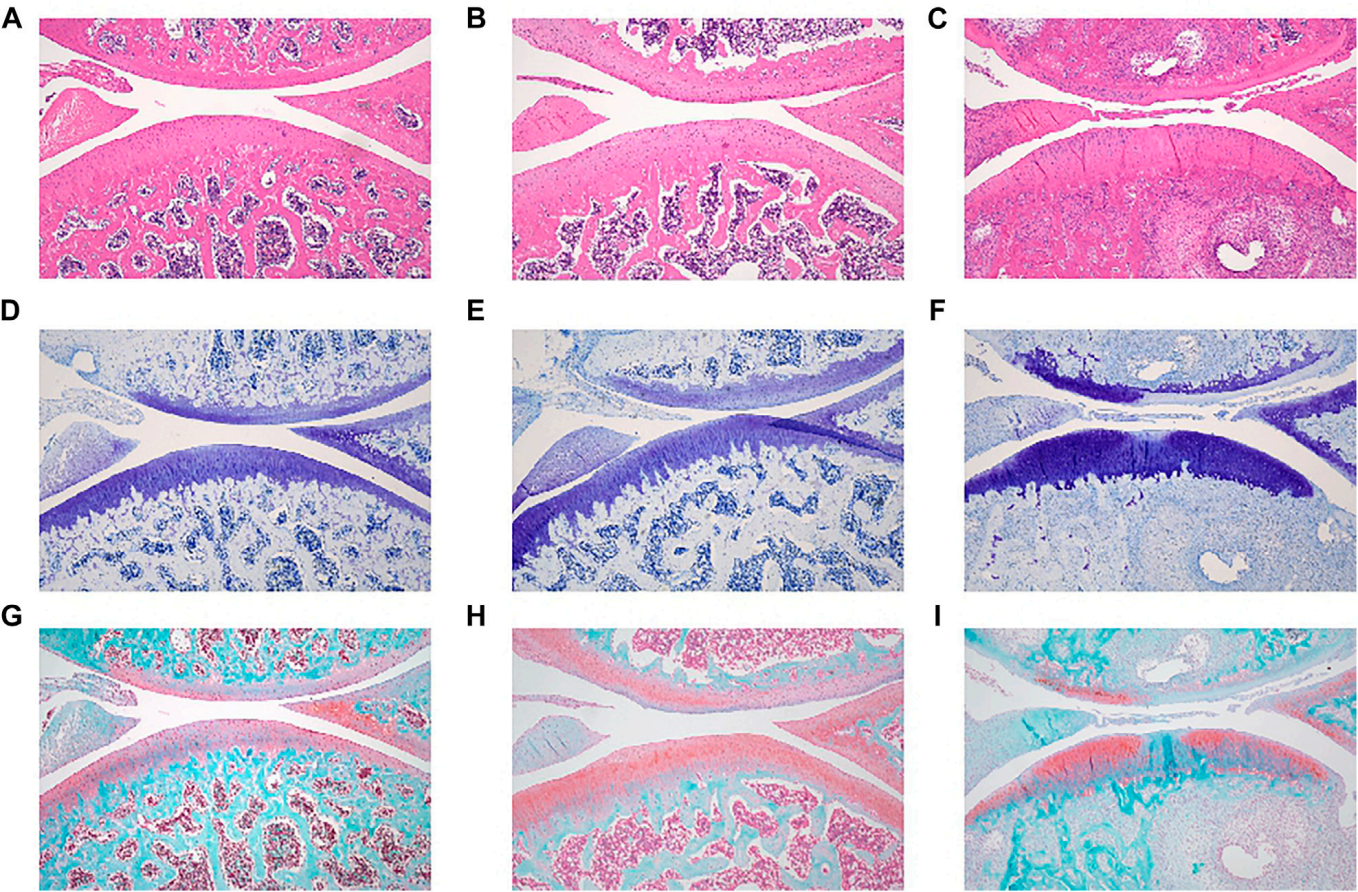
Representative microanatomical images of cartilage tissues in the different treatment groups showing HE staining, TB staining and SO/FG staining. HE staining of **(A)** control group, **(B)** OVX + ACLT, **(C)** PROTACs + ACLT. TB staining of **(D)** control group, **(E)** OVX + ACLT, **(F)** PROTACs + ACLT. SO/FG staining of **(G)** control group, **(H)** OVX + ACLT, **(I)** PROTACs + ACLT.

One month after ovariectomy and ACLT, the rats were euthanized, and the knee joints were evaluated by histological analysis. In the healthy group that underwent a sham operation, the cartilage layer of the knee joint was intact. The layer was clear, and the cartilage surface was smooth (Figure 4A). In contrast, ovariectomy and ACLT caused significant morphological changes in the cartilage, including 1) a rough cartilage surface, 2) a disordered cartilage layer, and 3) a thinned cartilage layer with small cracks (Figure 4B). Thus, a menopausal arthritis animal model was successfully established.

### Intra-Articular Blockade of Estrogen Function by Altering ERα Could Induce Cartilage Degradation

Results from frozen section of cartilage demonstrated the penetration of FITC-labelled peptide in cartilage tissue (Supplementary Figure S1). Then, we evaluated the efficacy of PROTAC-mediated blockade on cartilage repair and osteoarthritis (OA) progression. Rats were randomly assigned to 3 groups: one healthy group (sham operation) and two groups that were treated with OVX + ACLT and PROTACs + ACLT.

Rats were administered PROTACs intra-articularly once each week for four consecutive weeks and sacrificed in the fifth week. The joint tissues were harvested, and the sections were stained with HE, TB, and SO/FG. The stained slides were observed under a microscope, and images of the tissue sections were captured.

**FIGURE 5:**
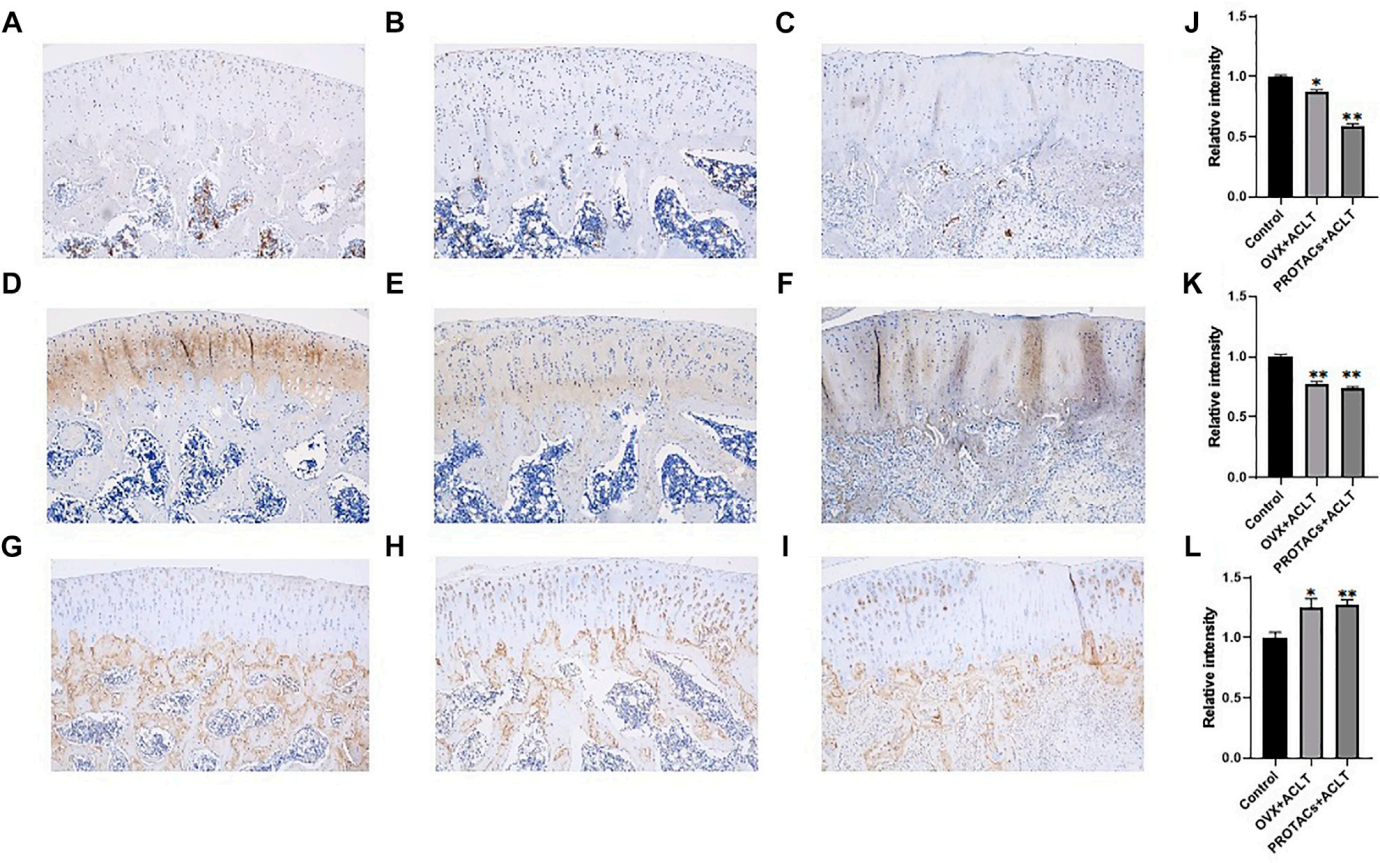
Representative immunohistochemical staining images of cartilage tissues in the different treatment groups. Scale bars, 250 μm. Each group contained 3 rats, and similar results were obtained in all rats. Anti-ERα immunohistochemical staining of **(A)** Control group, **(B)** OVX + ACLT, **(C)** PROTACs + ACLT. Anti-COL2 immunohistochemical staining of **(D)** control group, **(E)** OVX + ACLT, **(F)** PROTACs + ACLT. Anti-MMP-13 immunohistochemical staining of **(G)** control group, **(H)** OVX + ACLT, **(I)** PROTACs + ACLT. The intensity of immunohistochemical staining of ERα **(J)**, COL2 **(J)**, and MMP-13 **(L)**. The integrated optical density of immunohistochemical staining was quantified using the Image J software.

Semi-quantitative IHC results demonstrated that ERα expression in cartilage tissue was significantly downregulated after intra-articular PROTAC injection (Figure 5J). Consistent with the *in vitro* study results, this *in vivo* study results further supported the heterobifunctional peptide (TD-PROTAC) effect on ERα degradation, as indicated by previous research that PROTAC could selectively recruit ERα to the VHL E3 ligase complex, leading to the degradation of ERα in a proteasome-dependent manner (Jiang et al., 2018).

Significant cartilage damage was observed in the OVX + ACLT group. Morphological changes in the cartilage that are signs of OA have been described. As shown in Figure 3C, cartilage damage was present in the proteoglycan group after 4 weeks of PROTAC injection. Cartilage pathology was almost identical to that in the OVX + ACLT group (Figure 4B), as evidenced by a rough cartilage surface and disordered cartilage layer. This conclusion was further supported by the TB staining results.

TB is a metachromatic dye that stains proteoglycans in tissue because of its high affinity for sulfate groups. Significant loss or thinning of proteoglycans was observed in OVX + ACLT rat tissue (Figure 4E). Proteoglycan deposition in the PROTACs + ACLT group (Figure 4F) was similar to that in the OVX + ACLT group.

**FIGURE 6:**
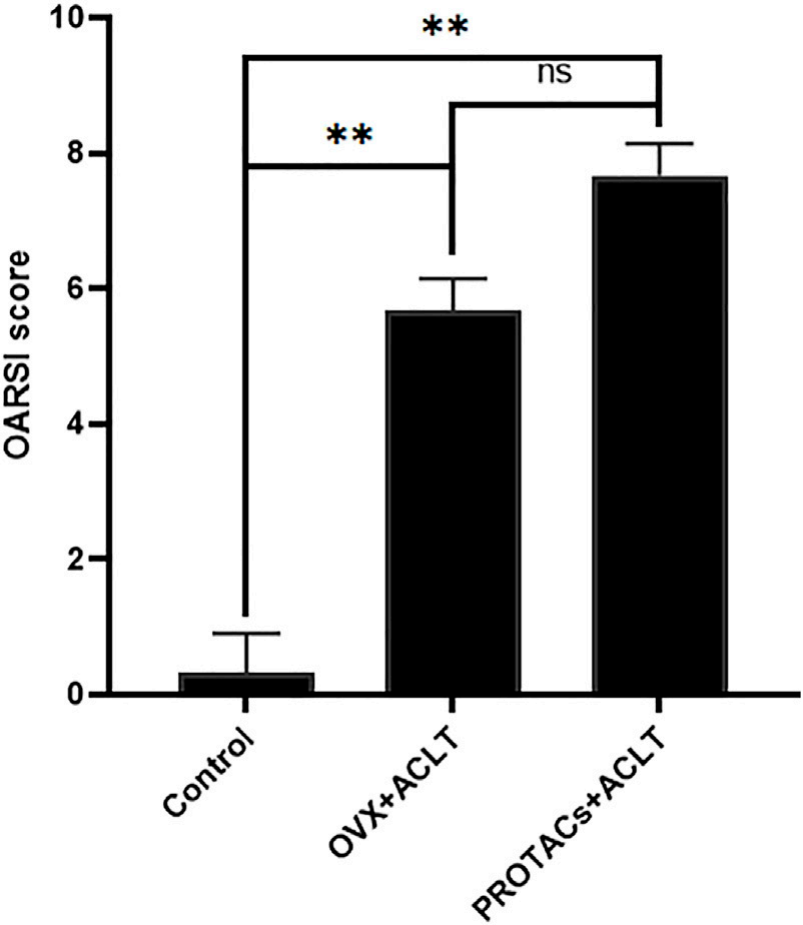
Histological scores of articular cartilage were quantitatively assessed based on the severity of cartilage damage. Cartilage destruction was scored in a blinded manner by 3 observers using the OARSI grading system.

Histological scoring was performed according to the modified OARSI assessment system. The degree of OA in HE- and TB-stained sections was scored by two investigators using an index combining the grade and stage (0–24 points). The results of the blinded assessment suggested that intra-articular delivery of PROTACs resulted in a score similar to that of ovariectomy. There was no significant difference between ovariectomy and intra-articular injection of PROTACs (Figure 6). These results further demonstrate the effect of PROTACs on blocking ERα *in vivo*.

The SO/FG staining results showed that the thickness of the proteoglycans in the PROTACs + ACLT group (Figure 4I) resembled that of the OVX + ACLT group (Figure 4H). Ovariectomy caused severe cartilage degeneration with profound loss of proteoglycan and cellularity compared to those in the healthy group.

Cartilage tissues were collected, and the expression of COL2 and MMP-13 was examined. As shown in Figures 5E,F,K, compared to that in the control group, both OVX + ACLT and PROTACs + ACLT treatment led to reduced protein levels of COL2 in cartilage tissue. MMP-13 expression levels were increased by OVX + ACLT and PROTACs + ACLT (Figures 5H,I,L). These findings were consistent with our previous study results showing that estrogen could inhibit MMP-13 expression.

Taken together, the results of this study suggest that intra-articular injection of PROTACs could significantly inhibit estrogen function and induce menopausal arthritis.

## Discussion

Because of the crucial role of estrogen deficiency in menopausal arthritis, the role of ERs needs to be defined (Roman-Blas et al., 2009). The most significant finding of this study is that we confirmed that ERα is one of the major ERs in joint cartilage matrix metabolism *in vivo*.

There are many ways to study the role of ERs in OA pathogenesis. Genetically modified animal models, such as knock-out or knock-in models, could be used for functional studies of ERs (Walker and Korach, 2004). However, it is time-consuming and expensive to establish a genetically modified animal model. To simplify the study process, small molecules or peptides that inhibit the function of the estrogen receptor function can provide a more convenient way (Speltz et al., 2018). In this study, we used a peptide to block the interaction between estrogen and ERα. This peptide, named PROTACs, can selectively recruit ERα to the VHL E3 ligase complex, leading to the degradation of ERα in a proteasome-dependent manner. The degradation of ERα could significantly enhance activities by reducing the transcription of genes downstream of ERα and inhibiting the proliferation of ERα-positive breast cancer cells, thus leading to *in vivo* tumor regression in the MCF-7 mouse xenograft model (Jiang et al., 2018).

The targeted proteolytic chimera (PROTAC) designs a specific ligand for the target protein of interest, connected to the E3 ubiquitin ligase ligand via a linker. PROTACs represent a chemical protein degradation approach. More and more proteins of interest have been successfully degraded by the PROTAC method for functional research and drug development (Itoh, 2018; Jin J. et al., 2020; Feng et al., 2020; Mukhamejanova et al., 2021). At present, PROTACs have unique advantages over classic inhibitors and are mainly used to discover new anticancer drugs. We found that applying this new strategy to achieve effective protein knockdown *in vivo* animal models is a unique model for studying specific signaling pathways. Therefore, PROTAC is expected to provide useful chemical knockdown tools for *in vitro* and *in vivo* research in a convenient, fast, controllable, and reversible manner (Sun et al., 2019a; Jin YH. et al., 2020; Feng et al., 2020).

In this study, we found that the degradation of ERα could significantly promote the expression of MMP-13, which could degrade COL2 and thus initiate OA. It has been demonstrated that the interaction of estrogen and ERα enhances miR-140 (an inhibitor of MMP-13) expression levels and thus protects cartilage from degradation induced by the inflammatory factor interleukin 1 beta (IL-1β).

Compared to the destructive role of estrogen-related receptor γ (ERRγ) in OA pathogenesis (Son et al., 2017), this study demonstrated the protective role of ERα in cartilage matrix metabolism by inhibiting matrix catabolism. Even though ERRγ is not a response element for estrogen, ERα and ERRγ exert opposite effects on cartilage matrix metabolism. This study provides novel evidence elucidating the function of E2/ERα in OA prevention.

## Data Availability

The raw data supporting the conclusion of this article will be made available by the authors, without undue reservation.

## Supplementary Material

Supplementary Figure 1
*In vivo* transfection efficiency analysis. Histological assay of FITC-labelled peptide uptake in cartilage tissue.
