# Supplementary figures and images for: ERα-Targeting PROTAC as a Chemical Knockdown Tool to Investigate the Estrogen Receptor Function in Rat Menopausal Arthritis
Source: Front Pharmacol. 2021 Nov 30;12:764154. doi: 10.3389/fphar.2021.764154 (PMC8669996; doi:10.3389/fphar.2021.764154)

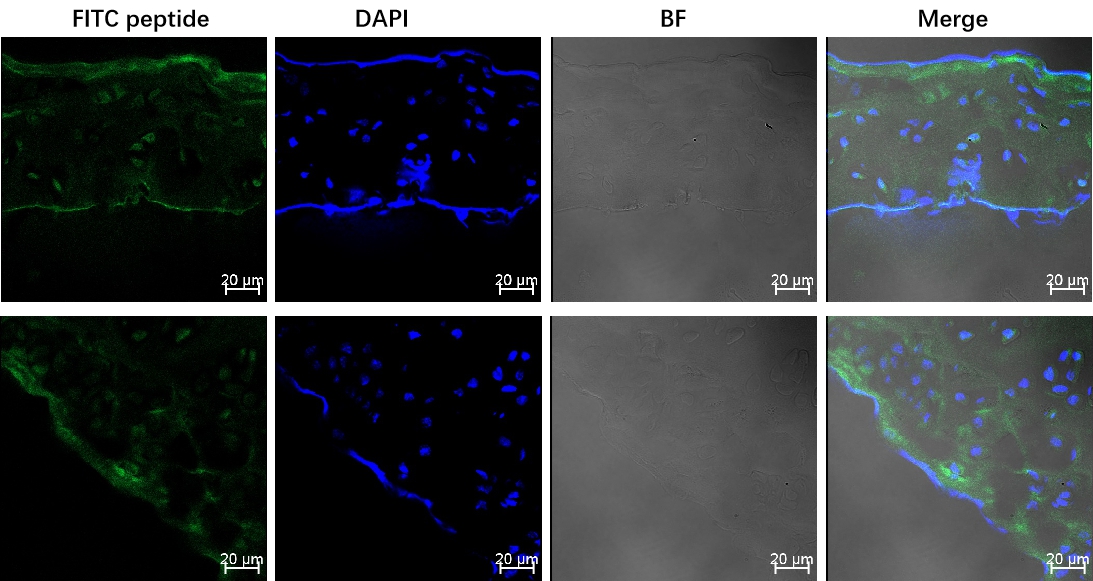

Supplement: Supplementary file 1 [file Image1.JPEG]
